# The Impact of Preoperative Corneal Epithelial Refraction Toricity on Transepithelial Photorefractive Keratectomy for the Treatment of Hyperopia or Mixed Astigmatism

**DOI:** 10.3390/vision9030057

**Published:** 2025-07-11

**Authors:** Diego de Ortueta, Samuel Arba-Mosquera

**Affiliations:** 1 Aurelios Recklinghausen, Erlbruch 34–36, 45657 Recklinghausen, Germany; 2 Faculty Medicine, University Navarra, 31009 Pamplona, Spain; 3 SCHWIND Eye-Tech-Solutions, 63801 Kleinostheim, Germany; samuel.arba.mosquera@eye-tech.net

**Keywords:** TransPRK, transepithelial photorefractive keratectomy, hyperopia, hyperopic astigmatism, mixed astigmatism, epithelium, epithelial toricity

## Abstract

This study analyzed the impact of corneal epithelial refraction on the correction of hyperopic and mixed astigmatism eyes treated with transepithelial photorefractive keratectomy. From the epithelial refraction provided by the diagnostic device, OCT correlations were evaluated with respect to manifest refraction. The postoperative outcomes showed a mean sphere of −0.03 D and a mean cylinder of −0.33 D, with 93% and 98% having 0.5 D, 1 D, or less spherical equivalent refractive error. The epithelium showed preoperative toricity: at 6 mm, the epithelium showed a compensational effect of ~15% for the refractive astigmatism, whereas at 3 mm, the compensation accounted for ~25% of the refractive astigmatism. No correlation was found between preoperative epithelial refraction and refractive deviation after hyperopic or mixed astigmatic transepithelial photorefractive treatment. This work provides insight into the refractive compensatory impact of the epithelium, suggests how one can benefit from that in transepithelial corrections, and sets a framework for the potential induction of errors in non-transepithelial corrections.

## 1. Introduction

Hyperopia and mixed astigmatism are refractive errors that can be treated with laser vision correction [1,2]. Both laser in situ keratomileusis (LASIK) [3,4,5,6] and PRK [7,8] are effective treatments for these conditions.

Transepithelial photorefractive keratectomy (TransPRK) is a recent variant of photorefractive keratectomy (PRK). The data for TransPRK with hyperopia are scarce [9]. The benefits of this procedure include a reduced surgical time, non-contact surgery with the corneal surface, faster surface healing and vision correction, and less postoperative discomfort and dry eyes [10]. We performed advanced surface ablation using the SCHWIND AMARIS platform [11]. Without breakthrough ablation in a reversal profile of the stroma and epithelium” reversed single step”, there is a sequentialization of the corneal aspherical profile: first, the calculated stroma is ablated; then, the planned epithelial profile is ablated without interruption.

The ablation rate varies between the epithelium and stroma [12]. The ablation of the epithelium is approximately 15% greater than that of the superficial stroma [13], resulting in potential inaccuracies if this discrepancy is not considered. The excimer laser loses energy in the periphery, exacerbating the issue if not addressed [14].

The importance of epithelial thickness (and associated refraction) has been reported by several studies [15], including by Reinstein et al. [16]. Using modern diagnostic systems with anterior optical coherence tomography, we can obtain epithelial maps and analyze the epithelium and stroma independently [17].

To the best of our knowledge, there is no previous work addressing this compensatory toricity of the epithelium to the manifest refraction in the setting of transepithelial ablations and the implications thereof.

In this study, we determined and evaluated the impact of the optical power of the corneal epithelium in relation to the manifest refraction. In particular, we evaluated the effect of epithelial toricity (i.e., the difference in curvature or optical power between the two perpendicular principal meridians) to determine whether epithelium power had an impact on manifest refraction. This may be relevant for all kinds of refractive corrections aimed at modifying the morphology of the cornea, as well as provide insights into previous observations regarding epithelial remodeling.

## 2. Materials and Methods

The records of 95 eyes of 66 patients who were consecutively treated with an aberration-neutral [18] TransPRK profile to correct hyperopia, with or without astigmatism and mixed astigmatism, were examined. TransPRK is an advanced method of surface treatment. The laser system thus ablates the regenerating surface of the eye, the epithelium, and then the stroma without interruption. Aberration-neutral profile [18] is an ablation technique that has been optimized so that it does not induce changes in the wavefront aberration other than the sphere and cylinder components. It is also multidynamic because it depends on several independent parameters (dimensions), including the sphere, cylinder, and optical zone size. The demographic data are presented in Table 1.

The preoperative refractive data and the epithelium map, which was measured using the CSO MS-39 (Construzione Strumenti Oftlamici, Firenze, Italy) optical coherence tomography with placido rings for the topography device, were included in the evaluation [19].

We analyzed the refractive outcomes, as proposed by the JRS [20]. Refractive and visual outcomes were analyzed using Excel software (Microsoft Corp.). The logMAR visual acuities were converted to Snellen acuities for data reporting using the Visual Acuity Conversion Chart of the Journal of Cataract & Refractive Surgery. The data entered into the laser system for refraction was the cycloplegic subjective refraction. For the preoperative epithelium map, we used the data from the MS-39 [21], we collected at least three images, and we only analyzed those of high quality. Using this device, we obtained the epithelium power for the sphere and cylinder at 3 and 6 mm, as given by the MS-39 software. After 4 months, we evaluated the refractive data again.

All procedures were conducted by a sole surgeon, DdO, using the SCHWIND AMARIS 1050RS [22]. The surgical procedure was described previously [23]. The diameter of the laser spot was 0.54 mm, and the optical zone used was at least 6.75 mm, with a multidinamic transition zone provided by the software as it depends on the sphere, cylinder and optical zone size. The AMARIS 1050RS uses the rotational symmetric ablation of the epithelium based on the parabolic fit of the individual epithelial thickness measured (provided) by the MS-39 OCT [24]; thus, it is customized, to a certain extent, based on the individual’s epithelium and rotational symmetric.

All eyes with a minimum follow-up of 4 months were included in the study; the exclusion criteria were eyes that had undergone previous ophthalmic eye surgery or that had diseases that could influence the healing of the cornea, such as systemic rheumatic or immunological illness, a preoperative central corneal thickness less than 440 microns systemic, a calculated postoperative corneal bed thickness less than 350 microns after ablation, previous ocular surgery, the asymmetric topography of 1 D or more, or keratometry higher than 49 D.

We retrieved the epithelial refraction from the measurement, considering the toricity for the cardinal and oblique astigmatism [25], to determine the relationship between this estimate and the manifest refraction.

The study was conducted according to the guidelines of the Declaration of Helsinki, and all the data were fully anonymized before we accessed them. Informed consent was obtained from all the patients. As the study was observational and retrospective, the Institutional Board decided that we did not need ethical approval.

## 3. Results

Table 1 reports the key refractive data. The average preoperatively treated sphere was +1.6 diopters (D), with a range of +0.25 to +6 D. The average cylinder measurement was −2.16 D; treatment extended to −5.75 D. Four months after TransPRK, the average manifest sphere was +0.13 D, and the manifest cylinder was −0.33 D.

**Figure 1:**
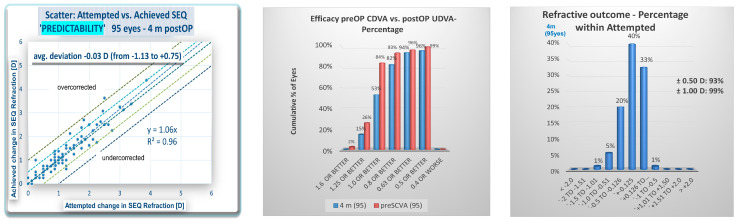

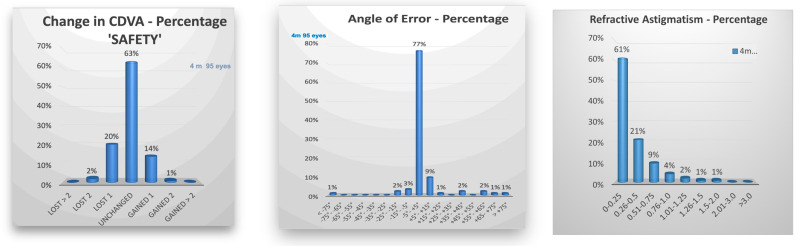
(**Top left**) histogram showing predictability and a regression coefficient near 1. Three eyes needed retreatment (the mean spherical equivalent (SEQ) was −0.03 diopters (D), with a range of −1.13 D to +0.75 D); (**top middle**) graphic showing efficacy comparing preoperative corrected distance visual acuity (CDVA) with postoperative uncorrected distance visual acuity (UDVA); (**top right**) the percentage of eyes among those attempted. Ninety-three percent of the eyes had a postoperative SEQ of half a diopter or less refraction; (**bottom left**) percentage safety. No eye lost or more lines of CDVA; (**bottom middle**) an angle of error of less than 15° in 89% of the eyes; and (**bottom right**) the postoperative refractive astigmatism, with 82% of eyes having half a diopter or less.

The refractive outcomes are reported in Figure 1. The predictability shows that 93% achieved diopters of half or less of the spherical equivalent and that 99% reached a value under 1 D. The efficacy was approximately 90%, while 82% exhibited astigmatism under 0.5 D and 95% under 1 D.

**Figure 2:**
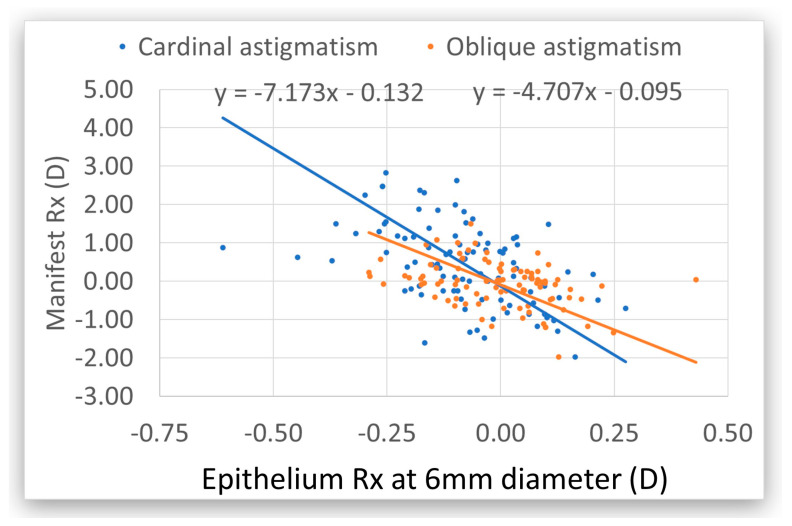
Preoperative epithelium refraction (Rx) in relation to the preoperative manifest refraction at 6 mm. The cardinal astigmatism is in blue and the oblique astigmatism is in orange. The manifest refraction is seven times higher than the epithelium Rx for cardinal and five times higher for oblique astigmatism. In both cases, the epithelium has toricity with the reverse sign, indicating a compensatory effect. At 3 mm, the epithelium compensates for 35% on both axes (Figure 3). The preoperative epithelium did not contribute to the manifest sphere. There was no correlation between postoperative manifest astigmatism and preoperative epithelium toricity.

Preoperatively, the cardinal astigmatism of the manifest refraction was seven times greater than the epithelial cardinal astigmatism at 6 mm. Oblique astigmatism of the manifest refraction was five times that of the epithelial refraction oblique astigmatism. The average for both axes was 5.8x, which means that the epithelium compensates for 17% of the refractive toricity at 6 mm (Figure 2).

## 4. Discussion

In this study, we calculated the impact of the optical power of the corneal epithelium on preoperative manifest refraction. We also calculated the toricity to determine whether epithelium power had an impact on manifest refraction. The toricity of the preoperative epithelium was correlated with preoperative astigmatism (accounting for 25–30% compensatory effect) but not with the postoperative one; no correlation was found with the sphere (neither pre nor postoperatively).

The preoperative epithelium does not contribute to the manifest sphere.

There was no correlation between postoperative manifest astigmatism and preoperative epithelium toricity, probably because the epithelium becomes more spherically shaped and has less toricity postoperatively.

We found that the postoperative manifest refraction is similar to the preoperative epithelium refraction. Thus, the preoperative epithelium might be one of the causes of undercorrection after hyperopic TransPRK; however, the analysis showed a high variance, with a correlation of only 0.32 (R2 < 0.1, i.e., >90% of the variance is unexplained by the correlation), which means that the epithelium is not the main cause of undercorretion in hyperopia after TransPRK.

Modern diagnostic devices provide more information about the epithelium. In our case, we demonstrated that the epithelium exhibits toricity preoperatively, with a reverse sign to the stroma. At 6 mm, the epithelium adjusts for astigmatism, which is around 17%; at 3 mm, this is 35%. The mean of both diameters is 26%.

We can estimate the spherical equivalent of the epithelium by averaging the axial power of all measured points (meridians) within a given diameter. Similarly, we can estimate the astigmatic components by subtracting the vertical from the horizontal meridians (cardinal astigmatism), and correspondingly for the oblique meridians (oblique astigmatism).

**Figure 4:**
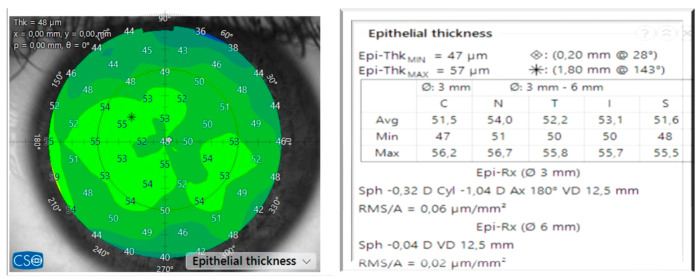
(**Left**)**:** epithelium map of a preoperative eye. (**Right**)**:** epithelial data as thickness centrally and maximally. Epithelium data at different positions in relation to the vertex of the cornea. Epithelium refraction at 3 and 6 mm.

Advanced anterior OCT devices measure the cornea and provide automatically computed information (Figure 4). This study employed this information to calculate the spherical component and astigmatism of the epithelium.

We employed a rotationally symmetrical profile for the epithelium using the SCHWIND AMARIS. The system adjusts the epithelium at the center and the periphery. It imports data from the center and the mean epithelium at a diameter of 3 mm and calculates the epithelium periphery with the mean epithelium thickness of the map between 3 and 6 mm. The system also considers energy loss at the periphery and increases the number of ablation spots at the periphery to offset the energy loss [14]. Thus, from the exported available epithelial map, the system performed a best fit of the parabolic central and peripheral epithelial thicknesses to obtain a rotational epithelial profile to model the ablation (and benefit from the masking effect of the epithelium).

Transepithelial approaches allow maximum correspondence between the corneal topography (as measured, i.e., including epithelium) and the ablation profile. Although there is a slight difference in the photoablative rates of the stroma and the epithelial tissue [13] (approximately 20% higher in the epithelium), the Amaris software compensates for this.

Epithelial irregularities (not captured by the fit, acting as a low pass filter), together with individual differences in the ablation rate, led to more stroma than necessary being ablated in some locations in all eyes. In contrast, in other locations, less epithelium than necessary might be ablated and some ablation corresponding to stroma would ablate the remaining epithelium.

In any case, there is some minor wasted tissue when the actual corneal epithelial profile is thinner than the applied epithelial ablation profile, and the stromal ablation is reduced when the actual corneal epithelial profile is thicker than the applied epithelial ablation profile. This seems to have no influence on the outcomes, as our results show.

If the epithelial profile from the center to the periphery is considered flat (equal thicknesses at all epithelial points, equivalent to a classic PTK ablation), a hyperopic shift of approximately 0.6 D can be expected [26].

The corneal epithelium plays an increasingly important role in corneal net power and, as a result, total ocular refraction. Epithelial refractive power is reported to be an average of 1.03 D (range 0.55–1.85 D) over the central 2 mm diameter zone and 0.85 D (range 0.29–1.60 D) at the 3.6 mm diameter zone [15]. In our case, we also demonstrated that the epithelium has an optical power with toricity that compensates for the manifest refractive astigmatism.

The advantage of treating the astigmatism with a rotational symmetric epithelium with TransPRK is that we treat the manifest astigmatism at the cornea (including its epithelium). If the epithelium is compensating for about 26% of the refractive astigmatism (which would be larger for the bare stroma), then the stroma astigmatism is 26% higher than the manifest astigmatism, which explains why PRK or LASEK could have undercorrections, as they are treated after we withdraw the epithelium; therefore, the toricity of the stroma is different. This is similar to LASIK or intralamellar surgery, where we treat the astigmatism without knowing the toricity of the epithelium (or that of the underlying stromal surface). Theoretically, some remodeling will occur after LASIK or intralamellar surgery, as the preoperative epithelium compensates for the preoperative refractive astigmatism.

The impact of the refractive power associated with the epithelial thickness in preoperative manifest refraction compensated for ~15% of the refractive astigmatism (12% for cardinal astigmatism and 18% for oblique astigmatism), whereas for 3 mm, the compensation accounted for ~25% of the refractive astigmatism (21% for cardinal astigmatism and 31% for oblique astigmatism). This means that the refractive astigmatism in the absence of epithelium (i.e., that of the bare stroma) would have been 18% and 36% larger at 6 mm and 3 mm, respectively. Accepting that 3–4 mm is closer to the pupil diameter for manifest refraction, it means that, for each diopter of refractive astigmatism, there is ~30% compensation by the epithelium, and the astigmatism at the bare stroma would be ~130% of the measured refractive astigmatism. Therefore, at least theoretically, all corneal refractive corrections that do not utilize transepithelial approaches like intrastromal, lenticlar, or surface ablation underestimate the level of refractive astigmatism.

A better analysis could have been conducted based on corneal and stromal toricity, not indirectly through manifest refraction; however, manifest refraction correction is the goal of all refractive therapies.

No correlation was found postoperatively. This appears to contradict the notion that the toricity of the preoperative epithelium (acting as a masking agent as demonstrated here) should be reflected in refractive deviations postoperatively [27]. This can be explained by several factors. On the one hand, we aimed to correlate preoperative epithelial toricity with postoperative manifest refraction (deviations). However, the refraction of the corneal (and potentially stromal) toricities is modified (all reduced) by the transepithelial treatment so that the correlation between them disappears [28]; in addition, epithelial remodeling over a less toric stromal surface would result in an even less toric postoperative epithelium. On the other hand, and contrary to common intuition, the application of a rotational symmetric epithelial profile may be an advantage (and not a limitation) of transepithelial ablations. This, if confirmed, would move away from the notion that a detailed epithelial map should be used for transepithelial ablations. The rotational epithelium and toricity are components of the manifest astigmatism that we treat with a transepithelial refractive correction.

Using a detailed epithelial map (assumed free of errors) would make transepithelial ablations the perfect laser PRK; however, it would suffer from the same underestimation of the refractive astigmatism at the stromal plane and would require the postoperative epithelium to again mask the toricity of the postoperative underlying stroma. Assuming the same level of postoperative compensation, an estimated 20% undercorrection in refractive astigmatism could be explained for surface ablations that are not transepithelial as PRK. However, empirical nomograms may have accounted for this, further reducing the differences between PRK and TransPRK, as compared previously [29].

A rotationally symmetric epithelial model (as used here, and presented elsewhere [30]) effectively shifts the already compensated (i.e., less toric and, in general, less aberrated) anterior epithelial surface to the anterior stroma and then applies the correction (measured for the anterior epithelial surface). Further, the reverse transepithelial approach (as used here and presented elsewhere [31]) reshapes the correction (measured for the anterior epithelial surface) at the already compensated (i.e., less toric and, in general, less aberrated) anterior epithelial surface, and the resulting (ideal) shape is then transferred to the anterior stroma by the rotationally symmetric epithelial model.

The error incurred is the mismatch between the detailed epithelial map (assumed free of errors) and rotationally symmetric epithelial model (as used here) multiplied by the mismatch between the ablation rates for the epithelium and stroma. Thus, a 20% mismatch in the epithelium and a 20% mismatch in the ablation rate would be masked by the epithelium remodeling, resulting in less than 3% induced error; therefore, the refractive result will be better if we use a transepithelial rotational approach instead of an epithelial map without knowing the stromal astigmatism.

Epithelial cardinal and oblique astigmatism (i.e., epithelial toricity) at diameters of both 3 mm and 6 mm are negatively correlated with manifest refractive astigmatism, suggesting a masking (partial) compensational effect, with a higher compensatory effect for the smaller diameter. Consequently, the larger the manifest cylinder, the greater the epithelial toricity with reversal sign. For example, −2.25 D manifest astigmatism has an epithelial compensation of +0.5 D. This is consistent with previous studies determined by direct imaging [32].

The limitation of this study is that it analyzes the epithelium changes after TransPRK without a comparison group of other refractive procedures, such as PRK, LASIK, or corneal lenticular surgery.

## 5. Conclusions

The preoperative toricity of the corneal epithelium is correlated with the amount of preoperative refractive astigmatism. No systematic correlation was found between preoperative epithelium and refractive deviations after hyperopia transepithelial treatments. Part of the success of TransPRK (as used here) may be attributed to the use of a rotationally symmetric aspherical epithelial model.

This work provides insight into the refractive compensatory impact of the epithelium, suggests how one can benefit from that in transepithelial corrections, and sets a framework for potentially induced errors in non-trasepithelial corrections.

## Figures and Tables

**Figure 3:**
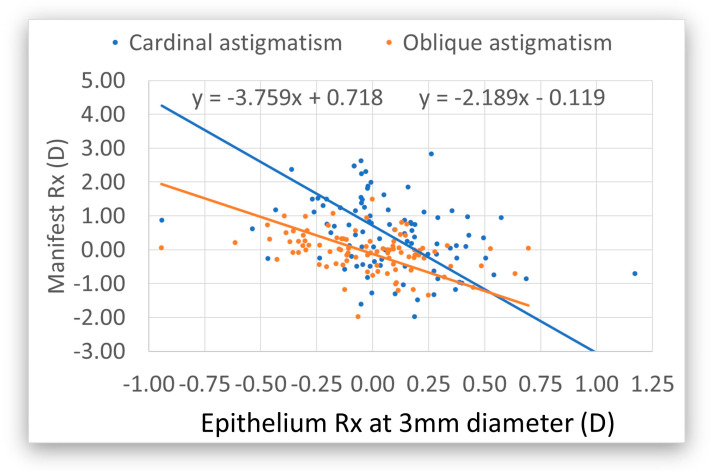
Preoperative epithelium refraction (Rx) in relation to the preoperative manifest refraction at 3 mm. In blue is the cardinal astigmatism. The manifest refraction is 3.8 times higher than the epithelium Rx. For the oblique astigmatism (in orange), the manifest Rx is 2.2 times higher than the epithelium Rx. In both cases, the epithelium has toricity with a reverse sign, indicating a compensatory effect. For cardinal and oblique astigmatism, the mean is 2.9x. The manifest astigmatism is higher, meaning that the epithelium compensates for 35% of the refractive toricity at 3 mm.

**Table 1:** Demographic data of the treated eyes and preoperative and postoperative refractive outcomes after 4 months. SEQ: spherical equivalent, Cyl: cylinder. Data are presented as mean ± standard deviation.

n = 95 Eyes				
Gender	Female	46.3% (44 eyes)		
	Male	53.7% (51 eyes)		
Age	36 years	(18–64 years)		
Eye	Left	51.6% (49 eyes)		
	Right	48.4% (46 eyes)		
	Preoperative	Range	Postoperative 4 m	Range
SEQ	+0.53 ± 34 D	(−1.75 to +3.88 D)	−0.03 ± 0.3 D	(−1.13 D to +0.75 D)
Sphere	+1.6 ±1.16 D	(+0.25 to +6 D)	+0.13 ± 0.29 D	(−0.5 D to +1 D)
Cyl	−2.16 ± 1.32 D	(−5.75 to −0 D)	−0.33 ±0.38 D	(−1.75 D to 0 D)

## Data Availability

All the data are fully anonymized and are available upon request.
